# VacQuant: a tool to quantify neurodegeneration and associated vacuolation in brain tissue

**DOI:** 10.1080/19336934.2025.2558387

**Published:** 2025-09-24

**Authors:** Kate L. Jordan, Colin D. Veal, Charalambos P. Kyriacou, Flaviano Giorgini

**Affiliations:** Division of Genetics and Genome Biology, University of Leicester, Leicester, UK

**Keywords:** Neurodegeneration, tau, *Drosophila*, fruit flies, vacuoles

## Abstract

Neurodegenerative diseases are devastating conditions characterized by progressive cognitive decline with few available treatments. Neurodegeneration can be quantified in vertebrate and invertebrate models of disease by analysis of vacuolation – the formation of empty spaces within brain tissue. Previous approaches for quantifying this phenotype have required time-consuming methods such as manual counting and measuring of vacuole dimensions, which can be subjective. Here we describe VacQuant, a novel application that can be paired with existing machine learning software to automatically measure the area of vacuolation in brain tissue. Using *Drosophila* brain sections from tauopathy model flies, a well-described model of dementia-related neurodegeneration, we quantified a significant increase in brain vacuolation at several timepoints in adult flies with the aid of VacQuant. When compared with quantification by five blinded volunteers, the machine learning method positively correlated with their group average, confirming its accuracy and functionality. This automated method developed with VacQuant removes human bias and measurement variation, providing a consistent threshold for all brain sections and experiments. This automated pipeline will be particularly useful for high-throughput screening for genetic modifiers or therapeutic compounds in animal models of neurodegeneration.

## Introduction

Neurodegenerative diseases are devastating conditions characterized by progressive cognitive and motor decline as a result of neuronal dysfunction or death with few available treatments. Most *in vivo* investigations into neurodegenerative disease mechanisms and novel therapeutic targets use mouse models before translation to the clinic. However, rodents are costly to maintain, have relatively long generation times and present significant ethical issues. *Drosophila melanogaster* provide an alternative model that is inexpensive, with a short-generation time, a superior genetic toolbox [1] and fewer ethical issues. ~75% of human disease genes have orthologues in *Drosophila* [2] with all the key human physiological processes conserved in flies. In addition, human disease genes such as those associated with neurodegenerative disorders that act in a gain-of-function manner can be expressed in flies, resulting in phenotypes that are comparable to human patients such as reduced lifespan, motor impairment or cognitive defects [3]. Notably, *Drosophila* is very amenable for both genetic and candidate drug screening, thereby facilitating the drug development pipeline for these disorders.

A feature of neurodegeneration in humans, as well as animal models, is the formation of visible, empty cavities in the brain known as vacuoles, formed due to cell loss. This is exemplified in fruit flies that express the R406W mutant form of tau throughout the nervous system [4], which serve as a model for frontotemporal dementia and parkinsonism linked to chromosome 17 [5]. Tau is a microtubule-associated protein that provides structural stability and contributes to axonal transport. Hyperphosphorylation and aggregation of tau into fibrils and paired helical filaments is a primary hallmark for several neurodegenerative diseases collectively known as tauopathies [6]. While healthy control flies exhibit age-related vacuolation due to normal ageing, a dramatic increase in vacuolation is observed in flies expressing tau R406W throughout the nervous system [4]. Pan-neuronal overexpression of wild type (WT) tau also leads to a vacuolation phenotype, but to a much lesser extent which is less associated with ageing.

Current methods for vacuolation quantification often require manual scoring of vacuoles either by counting the number present in a section, and/or measuring the area or diameter of the vacuole using imaging software [4,7]. To quantify the size of the vacuoles Image J has been used to manually measure and count the number of vacuoles, categorizing them by size. The numbers of vacuoles in each category can then be divided by the number of sections to calculate the average per section [8]. This manual method for measuring individual vacuoles is both time-consuming and error prone. To mitigate the significant time required for this method, others have limited their quantification to only certain brain areas of interest, but this is not appropriate in cases where the phenotype is mild or widespread [7]. Another approach proposed a ’neurodegeneration index’ from 0–5 as a scale of increasing vacuolation [9]. Brain sections were observed and ranked based on representative images for each category, but the qualitative nature of this method makes it subjective and likely to vary substantially among researchers.

To reduce human error resulting from manual quantification methods, an advanced analytical method using optical projection tomography to image the *Drosophila* brain in 3D exists, which detects vacuoles by automatically measuring areas of fluorescence within a whole fly using MAPaint software. This method requires the exoskeleton of the fly to be cleared and bleached for imaging to allow full transmission of white and fluorescent light into the tissue [10]. Whilst this has advantages over manual quantification as the vacuoles can be imaged and analysed in 3D, it requires specialist microscopy to obtain the images, making it a costly alternative.

Here, we describe VacQuant, a novel ImageJ macro that when combined with existing machine learning software, overcomes the need for specialist equipment and improves quantification consistency by creating a standard threshold of measurement, as well as minimizing continual user input by automating the processing of bulk images. Together, this novel approach combines well established, convenient histological methodology with newer machine learning technology to create an efficient and accurate workflow for measuring vacuolar-mediated neurodegeneration in brain tissue.

## Materials and methods

### Fly husbandry

Vacuolation was characterized in *Drosophila* expressing mutant tau R406W and compared to *Drosophila* overexpressing tau WT, and control flies. All fruit fly stocks were maintained on standard maize food at 18°C in a 12-hour light/dark cycle. The *w,elavGAL4; +; + (c155)* stocks were obtained from the Bloomington Fly Stock Centre in Indiana. The following stocks were kindly provided by the Feany lab: *w; +; UAS-TauR406W; w; +; UAS-TauWT*. The *w; +; UAS-LacZ* line was kindly provided by Alf Herzig. All experiments and crosses were conducted at 25°C. The UAS/GAL4 system was utilized to drive expression of the transgenes pan-neuronally using *elavGAL4*. The transgenic fly lines of interest (LacZ expression control, tau WT, and tau R406W) were crossed with the *elav* driver to obtain the experimental females. The flies were aged to days 10 and 30 to chart the age-related progression of the phenotype.

### Preparation of *Drosophila* brain sections for analysis

Experimental flies were fixed for 24 hr in 10% formalin and then embedded in paraffin wax. Whole flies were embedded to facilitate orientating the specimen, as opposed to embedding the small fly head alone, which was difficult to orientate correctly. Once embedded, the fly blocks were serially sectioned at 3 µm thickness and stained with H&E following standard protocols [11]. Once the tissue was sectioned, all sections were imaged at 20x magnification on a Nikon Eclipse Ci-L equipped with the DS-Fi3 digital camera, which has a 5.9 megapixel CMOS image sensor allowing for the capture of images up to 2880 × 2048 pixels.

### Quantification of vacuolation

This novel approach pairs a protocol to train vacuole detection and measurement using the Trainable Weka Segmentation (TWS) plugin included in the image processing package, Fiji [12], with the VacQuant macro to automate the quantification of large sample groups. The source tissues utilized for development of VacQuant were brain sections from *Drosophila melanogaster* expressing either tau WT or tau R406W throughout the adult nervous system. To differentiate and measure the vacuoles from surrounding brain tissue, the TWS plugin utilizes the random forest machine learning method to provide an accurate classification of the image [12]. The classifier is trained using a set of images to classify the pixels within an image into predetermined segments or classes. TWS provides many options for customizing the training and classification features, including Gaussian blur, Sobel filters, etc [12]. For the identification of vacuolation, edge detection features (Sobel filter, Hessian, and Difference of Gaussians) were chosen to identify the borders of the vacuoles and brain tissue, and the border of the brain tissue and surrounding white background. These features allow for identification of objects and areas of differing contrast and intensity, and classification of the bordering pixels on the limits of the vacuoles or brain tissue [12]. Guassian blur was also selected as a noise removal feature to reduce the noise and intensity variation from the staining process [12]. Finally, the membrane detection feature ‘Membrane projections’ was selected to improve the signal of the borders and reduce noise from varying levels of intensity across them resulting from the staining [12].

The method used to train the classifier is described in detail in Figure S1. For optimal performance, it is recommended that each user employs a sampling of their own images to train the classifier to ensure it is tailored to the level of staining and tissue type. If the tissue preparation between experiments is consistent, it is only necessary to train the classifier once. If the tissue preparation varies greatly, it is recommended to train the classifier for each experiment to ensure the same threshold of detection and sensitivity is applied to all genotypes. It requires the manual labelling of a random sample set of images to identify which pixels must be classified into each category (background, brain tissue, vacuoles). The number of labels required for an accurate quantification varies per image but was approximately 3–6 per image, providing one or two examples for each category. Once the training process has been repeated on several images to reach a quantification the user deems to be accurate, the classifier can then be applied to other images to automatically quantify the image.

**Figure 1.**
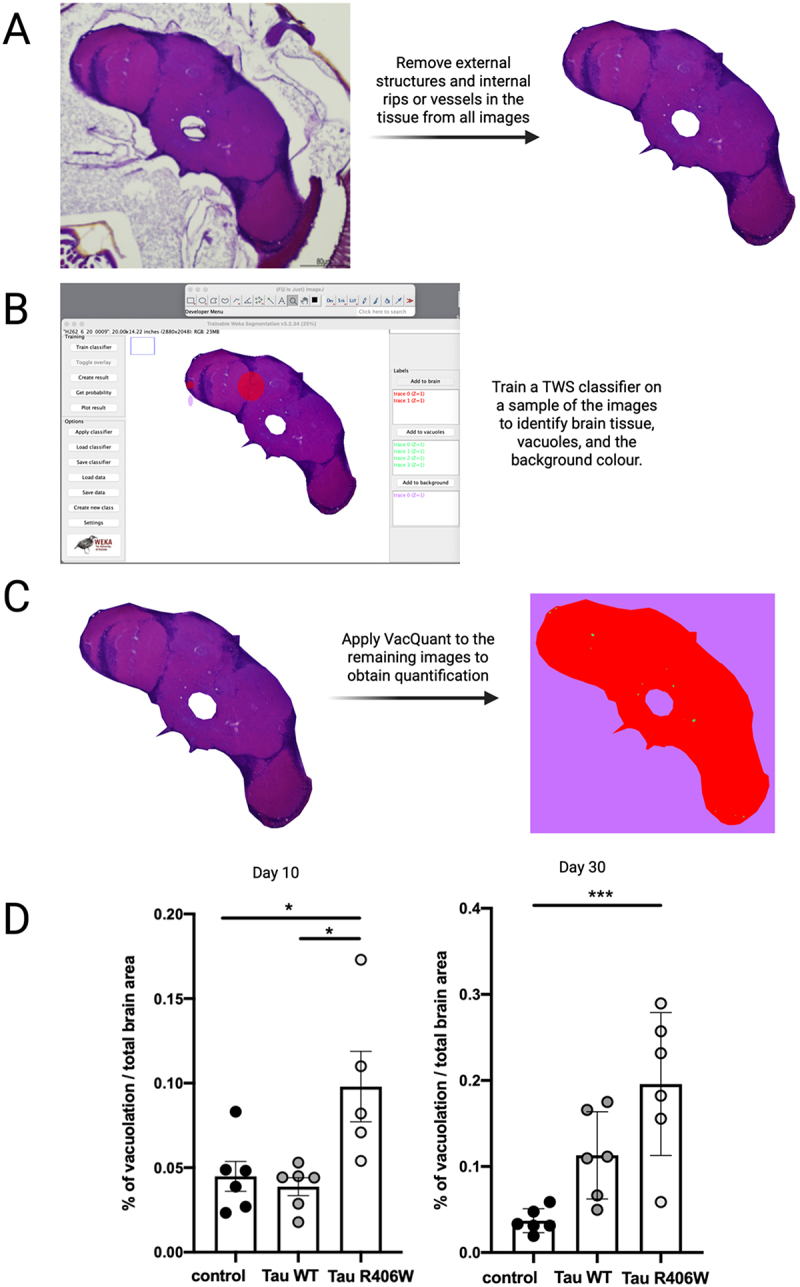
Workflow for assessing vacuolation using VacQuant. (A) Images of *Drosophila* brain tissue were cropped to remove external structures, internal rips or vessels in the tissue prior to the application of the trainable weka segmentation (TWS) plugin to ensure they are not erroneously classified as vacuoles or brain tissue. Scale bar = 80 µm. (B) The trainable weka segmentation (TWS) plugin in Fiji can classify and segment pixels within an image to quantify internal structures. In this case, it was applied to images of *Drosophila* brain tissue to quantify vacuolation. A classifier must first be trained to recognise vacuoles, brain tissue and the image background using a sampling of images. A more detailed method of this training process is described in Figure S1. (C) the VacQuant macro was applied to all remaining images to obtain the quantification. Vacuoles are classified in green, brain tissue in red, and the background in purple. (D) Expression of tau R406W in *Drosophila* exhibits significantly higher levels of vacuolation than expression of WT tau, or control flies. Fly brains were fixed and processed at 10 days post-eclosion (Left panel) and 30 days post-eclosion (Right panel). Significance was calculated using a one way ANOVA and Tukey post hoc test. Left panel) F(2, 14) = 1.618, control vs WT tau *p* = 0.0237, control vs tau R406W *p* = 0.0122. Right panel) F(2, 15) = 4.184, control vs tau R406W *p* = 0.0006, *p < 0.05, ***p < 0.001. *n* = 5–6.

In this case, the classifier was trained on 10 images. Once the training was completed, the classifier and the data produced by the training process was used to classify experimental images and quantify the vacuoles and brain tissue. The classifier was trained to ignore varying textures in the tissue, only classifying empty spaces that pass all the way through the tissue to ensure stringency. However, when staining with H&E, many other structures aside from the brain tissue are also stained, such as the eyes. Because of this, the TWS plugin was unable to differentiate between brain tissue and other tissues that are not of interest. To circumvent this issue, the images were cropped using Fiji to remove other structures from the image leaving only the relevant brain tissue (Figure 1(A)). While this step is somewhat time consuming (approximately 20 seconds to 1 minute per image), it allows for human input prior to the machine learning quantification and any internal tears or rips in the tissue can be removed, so they are not confused for vacuoles during the classification step, making the quantification much more accurate and consistent. Once all images were cropped in Fiji, the machine learning plugin was applied.

### Automation of the quantification

While the classifier speeds up the quantification of individual images, manually loading the image, applying the classifier, and saving the data image by image is still time consuming. Because of this, a macro was encoded to automate the process using Fiji (Figure 1(C)). The macro outline is listed below:

1. Create a table to record the data
2. Use a loop function to iterate through the images
3. Open the first image
4. Open the plugin
5. Load the classifier and data
6. Classify the image
7. Select the generated classified image and save it in a separate folder
8. Open the histogram and record the pixel counts in each class to the results table
9. Calculate the percentage of vacuolation in the brain section
10. Close all windows and repeat
11. Save the quantification table when all images have been classified.

The macro saved the classified image under the original image name in a separate quantification folder so that the classification can be checked by the user for accuracy. The pixel counts were collated into a table and saved in the same location for subsequent statistical analysis. As macros in Fiji do not automatically wait at each step until the function is completed, several wait functions were added to ensure the task has been completed prior to the programme continuing, otherwise errors were generated.

## Results

### Case study

This method was tested using brain sections from flies expressing either tau WT or tau R406W throughout the adult nervous system at days 10 and 30 post-eclosion. Example images of control and tau R406W expressing flies demonstrating the accuracy of the pixel classification produced by the TWS classifier are shown in Figures S3 and S4 in the Supplementary Data. At day 10, tau WT expressing flies do not show significant levels of vacuolation as compared to the background levels of control flies. There was also no change in vacuolation levels observed between day 10 and 30 in the control flies. However, expression of tau WT leads to a 3-fold increase in vacuolation by day 30, suggesting an age-related phenotype. Meanwhile, expression of tau R406W leads to a significant increase in vacuolation as early as day 10 when compared to control and tau WT expressing flies. By day 30, tau R406W flies also show a 3-fold increase in vacuolation from day 10 levels, which is again significantly higher than control flies. Additionally, tau R406W flies also show a 2.5-fold increase in vacuolation when compared with tau WT flies, although it is not statistically significant. These results clearly demonstrate the mild phenotype observed in flies expressing tau WT, and by comparison, the severe vacuolation phenotype exhibited by flies expressing tau R406W (Figure 1(D)). These results validate previous findings and demonstrate the accuracy and functionality of this novel method [4]. We have performed further validation of the VacQuant pipeline via a detailed comparison of the approach with manual quantification by five blinded volunteers (see below), and found a strong correlation between the two quantification methods, while highlighting the reduction in variability when using our automated pipeline.

### Validation of the VacQuant application pipeline

**Figure 2.**
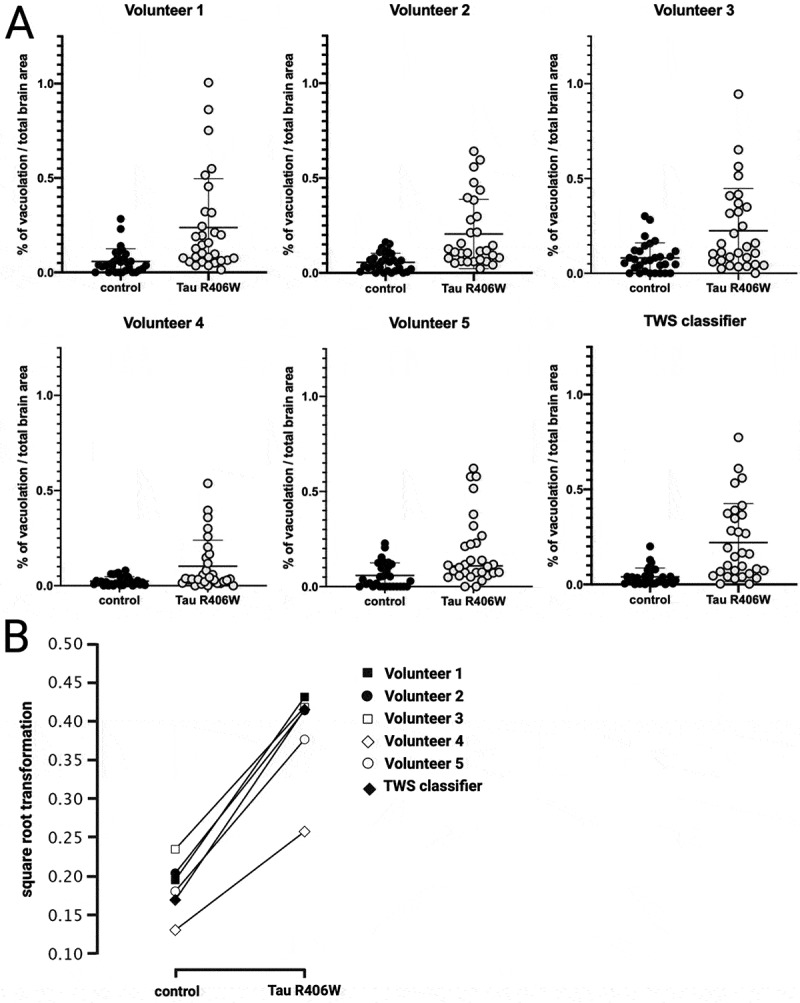
A) Five volunteers manually quantified the % area of vacuolation in *Drosophila* brain sections, whilst the trainable weka segmentation (TWS) machine learning classifier automatically quantified the brain sections. B) Square root transformation of the raw data was performed to normalise the data distribution. A two-way ANOVA on this transformed data revealed significant genotype (F_1,348_ = 114.2 ƞ2 = 0.234 (*p* <<< 0.001) and volunteer/classifier (F_5,348_ = 4.37 ƞ2 = 0.045 *P* < 0.001) effects, but no interaction (F_5,348_ = 0.87, ns).

To validate the VacQuant quantification pipeline, five blinded volunteers completed a manual quantification of a sample set of data from control flies and tau R406W expressing flies processed at day 30 post-eclosion. The volunteers were PhD students studying neurogenetics and were provided with several examples of vacuolar structures prior to quantifying the brain sections. The brain section images were anonymized, and they were not provided with information on the genotypes of the flies to ensure they were not biased towards quantifying more or fewer vacuoles. Thirty sections were randomly selected from each genotype, sampling five sections per fly. The five volunteers traced around the brain section in each image and measured the area of every vacuole they observed using Fiji to calculate the % of vacuolation in each brain section. A two-way ANOVA after square root transformation of the raw data revealed significant Genotype (F_1,348_  = 114.2 ƞ2 = 0.234 (*p* << < 0.001) and Volunteer/Classifier (F_5,348_  = 4.37 ƞ2 = 0.045 *P* < 0.001) effects, but importantly, no interaction (F_5,348_  = 0.87, ns) (Figure 2(A,B)). The significant Volunteer/Classifier effect was generated by the consistently lower scores of Volunteer 4 (Figure 2(B)).

**Figure 3.**
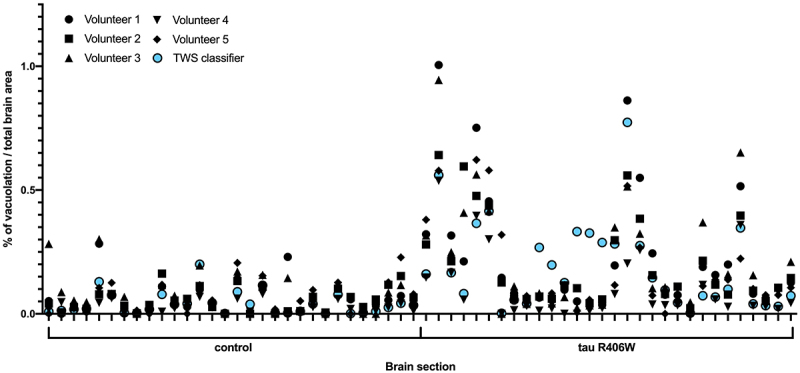
A comparison of five volunteers and a trainable weka segmentation (TWS) machine learning classifier quantifying vacuolation in *Drosophila* brain sections of control flies and tau R406W expressing flies fixed at day 30 post-eclosion. There is a greater level of variation observed between volunteers quantifying the tau R406W expressing flies when compared with control flies.

In the tau R406W brain sections, where there is a significantly higher level of vacuolation, there is also more variation among volunteers (Figure 3). This suggests there is a higher chance of missing vacuoles through human error when there are many more present. This would be exacerbated in cases when the phenotype consists of mainly very small and dispersed vacuoles. Indeed, for tau R406W brain sections in particular where the TWS classifier detected a higher % of vacuolation than the volunteers, it was observed that alongside larger vacuoles than observed in the control flies, these tau R406W brain sections also had a large number of small, disperse vacuolar structures that would be challenging for a human to quantify, yet the TWS classifier was able to accurately detect and quantify these. This wide variation among volunteers suggests that using this manual method does not provide a reliable quantification of the vacuoles. The machine learning algorithm removes this variability by providing a consistent threshold, which is generally within the ranges of the volunteers’ results.

**Figure 4.**
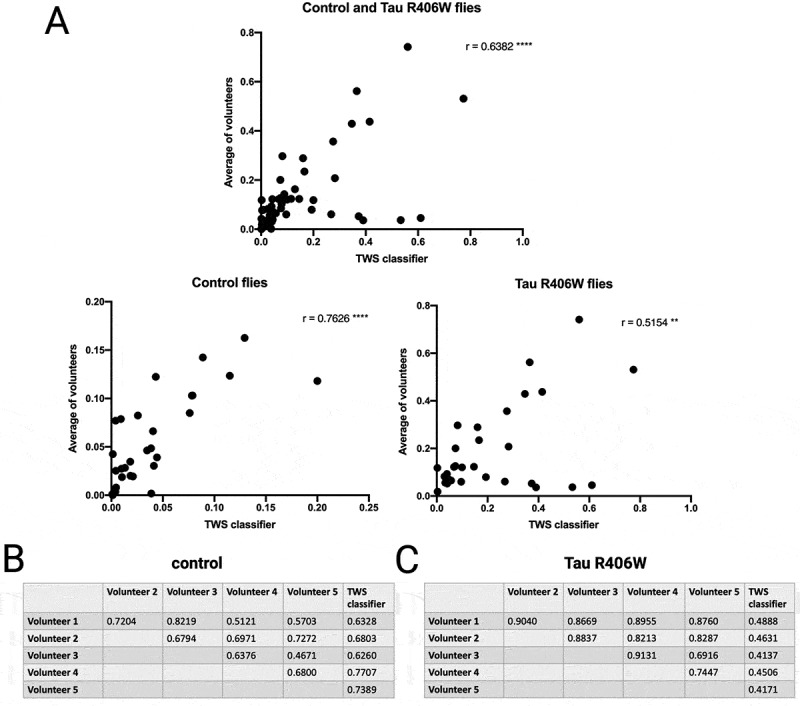
The average of five manual quantifications positively correlates with the TWS machine learning classifier quantification. This correlation was calculated for the control and tau R406W expressing flies collectively and separately. A) Control plus tau R406W flies ** *p* < 0.01 **** *p* < 0.0001. Correlation matrices for each volunteer/classifier for control and tau R406W flies are shown in panels B and C respectively. Critical *R* values are 0.317 and 0.437 at *p* = 0.05 and 0.01 respectively (28 df). All correlations are *p* < 0.05 and all but two, *p* < 0.01.

Correlation coefficients were calculated to compare the TWS classifier with the average of the five volunteers (Figure 4). Overall, there is a highly significant correlation between the human average and TWS classifier across both genotypes (*r* = 0.638, *p* < 0.001), further validating the machine learning algorithm. The correlation is stronger for the control flies (*r* = 0.763 *p* < 0.001) than the tau R406W expressing flies (*r* = 0.515 *p* < 0.01). However, as previously discussed, this is probably because the TWS classifier is able to quantify smaller vacuoles overlooked by humans, resulting in a higher quantification by the TWS classifier than the volunteers. Furthermore, correlation matrices among volunteer/TWS are highly significant for all comparisons for both genotypes (Figures 4(A,B)). For control flies, the correlation between each volunteer and TWS is particularly impressive (all *p* < 0.001), whereas correlations among volunteers, while always significant, were more variable (Figure 4(B)). Conversely, for tau R406W expressing flies, the inter-volunteer correlations were considerably higher (r, 0.69–0.91) than the correlations between each volunteer and TWS (r, 0.41–0.49) (Figure 4(C)). It is possible that the human volunteers consistently quantify the larger vacuoles present in these brain sections with accuracy and reliability, as they are easy to identify and trace, but they miss the smaller vacuoles also present that are detected by the TWS classifier.

The labour required to have multiple volunteers (at least 2) measure the vacuoles in each brain section by hand is considerably greater than running the TWS classifier (days versus hours). Consequently, the TWS classifier represents a valid, reliable, efficient and accurate machine learning algorithm for quantifying vacuolation in fly brains, which provides consistent results that are comparable to those from five manual quantifications.

## Discussion

This study describes a novel method to quantify vacuoles within brain tissue of *Drosophila* stained with H&E that is accurate and efficient. The machine learning software classifies pixels from a histological image in order to quantify vacuoles and represents a significant improvement upon previous methods requiring manual quantification. As the TWS plugin generates a classifier that can be used repeatedly to quantify the images, it provides a standard threshold by which all genotypes and samples are measured, improving consistency and removing the need for blinding the experiment from the user. While manual quantification has the potential to vary from user to user, especially at higher levels of vacuolation (Figure 3), the TWS classifier correlates well to the average of several volunteers, arguably making it more accurate than relying on a single scorer alone (Figure 4). Although the training of the TWS classifier does require manual input, once generated, application of the TWS classifier is user-independent. While cropping the image prior to the quantification is the most time-consuming part of this method, user input at this step allows for the removal of rips or tears in the tissue, which would otherwise affect the quantification of the brain area or be mistakenly identified as vacuolar (Figure S5). This refinement of the images ensures VacQuant produces the most accurate and stringent quantification and that damaged brain sections can still be quantified, reducing wastage. Where the phenotype is very strong and there are many vacuoles to be quantified, the TWS classifier greatly reduces the time taken for analysis. Additionally, the TWS classifier can be running in the background allowing the user to work on other tasks and can be run locally on a standard computer without the need for specialist servers or equipment. Classification of approximately 100 images may take up to an hour, but this is highly dependent on the speed of the computer being used. Finally, this method can be conducted on images taken with a standard light microscope and thus does not require specialist equipment, making it accessible to all laboratories. TWS also has many features that are customizable, making it applicable to many different quantifications and internal structures in *Drosophila* and other organisms.

VacQuant quantifies vacuoles as a percentage of the total brain area, which differs from other manual methods which use the vacuole number or diameter to provide a measure of vacuolation. In tissues where the vacuoles are small and numerous, the diameter size would not necessarily be representative of the full scale of the degeneration present in the tissue. Likewise, in tissues where there are a few large vacuoles compared to control lines with smaller vacuoles, it would not be representative of the scale of degeneration to only consider the number of vacuoles present. Indeed, within the same brain, there can be a variety of small and large vacuoles throughout the sections and therefore quantifying the number or diameter of these could result in highly variable results between sections. Hence, VacQuant determines the percentage of vacuolation in the total brain tissue as the most representative overall quantification of the amount of degeneration in the brain. Of course it is likely that, if the user required, further development of this method could be conducted to also quantify these additional parameters. Importantly, the size of the vacuole does not affect the success of detection by the classifier and it is capable of detecting large or small vacuoles (Figure S5).

All that is required for a successful quantification is a high contrast border between the objects/regions to be classified. Brain sections with lighter, less consistent staining were more likely to have mistakenly detected vacuoles during classification of the pixels due to the close colour value of the pixels to the vacuoles (Figure S5). Whilst this is a limitation, it can be easily overcome and was not a common issue during this project, as the majority of brain sections utilized during the experiments achieved the level of stain required for reliable and accurate quantification. If a high contrast is present in a specific tissue section, the classifier can be trained on any set of images from any model for any experiment. This adaptability of the plugin greatly increases its usefulness and could be applied to a wide range of projects. Aside from classifying vacuoles, the plugin could also be trained to recognize the formation of plaques or inclusions stained via immunohistochemistry to quantify other disease phenotypes. The macro can similarly be adapted to suit the user’s individual needs, selecting the images from their own computer files and saving the quantification wherever the user requires. As a result, this method may be utilized to quantify a wide range of structures or objects within high contrast biological sections to provide a quick and consistent measure. By saving the classified images, the quantification can be double checked by the user, providing an essential step in quality control.

With the lack of therapeutic compounds available for neurodegenerative diseases, the importance of high throughput screens of potential genetic modifiers is clear. However, with large scale screens, small effects can be overlooked, and possible therapeutic targets could be missed. As modifiers of disease often do not impact all disease phenotypes equally, a positive candidate could be omitted from further research if it generated a modest and varied effect during the selection process. Using a standard threshold created by machine learning reduces variation when compared with quantifications completed by observers, resulting in a more reliable and consistent measurement, enhancing statistical power to identify small positive effects of therapeutic compounds. It is important for promising avenues to be investigated thoroughly and so tools that make therapeutic screens more robust and sensitive are beneficial.

A troubleshooting guide is provided in the Supplementary Methods outlining some common issues encountered during this project and likely solutions for potential users.

## Supplementary Material

Jordan et al Methods and Technical Adv Supplemental clean.docx

## Data Availability

All data contained herein is available upon request from the corresponding author. The code for the VacQuant, an ImageJ (https://imagej.net) macro is available on GitHub https://github.com/colinveal/VacQuant
