# Supplementary material for: VacQuant: a tool to quantify neurodegeneration and associated vacuolation in brain tissue
Source: Fly (Austin). 2025 Sep 24;19(1):2558387. doi: 10.1080/19336934.2025.2558387 (PMC12461890; doi:10.1080/19336934.2025.2558387)
Supplement: Jordan et al Methods and Technical Adv Supplemental clean.docx [file KFLY_A_2558387_SM0384.docx]

**Supplementary Data
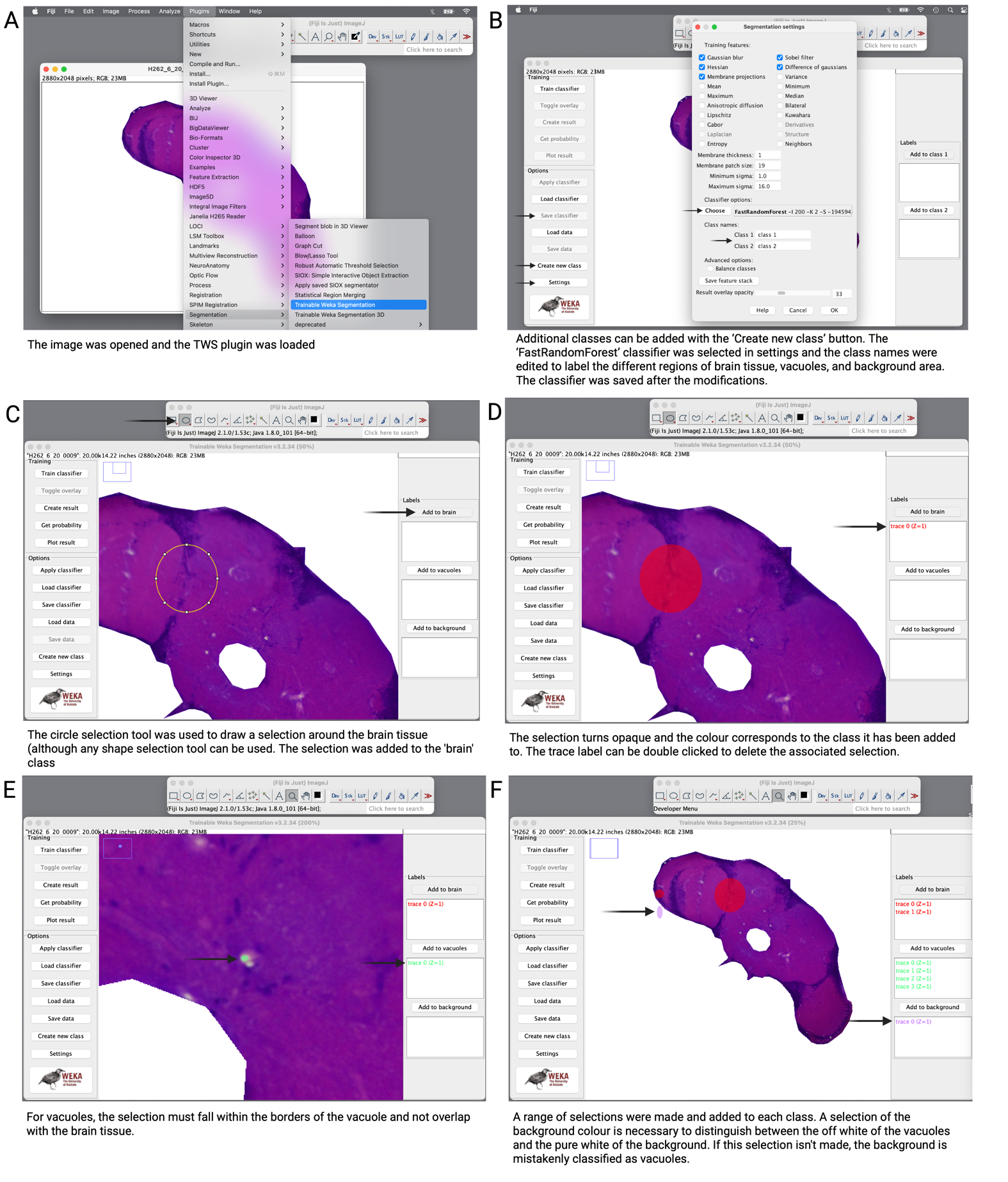
**

**
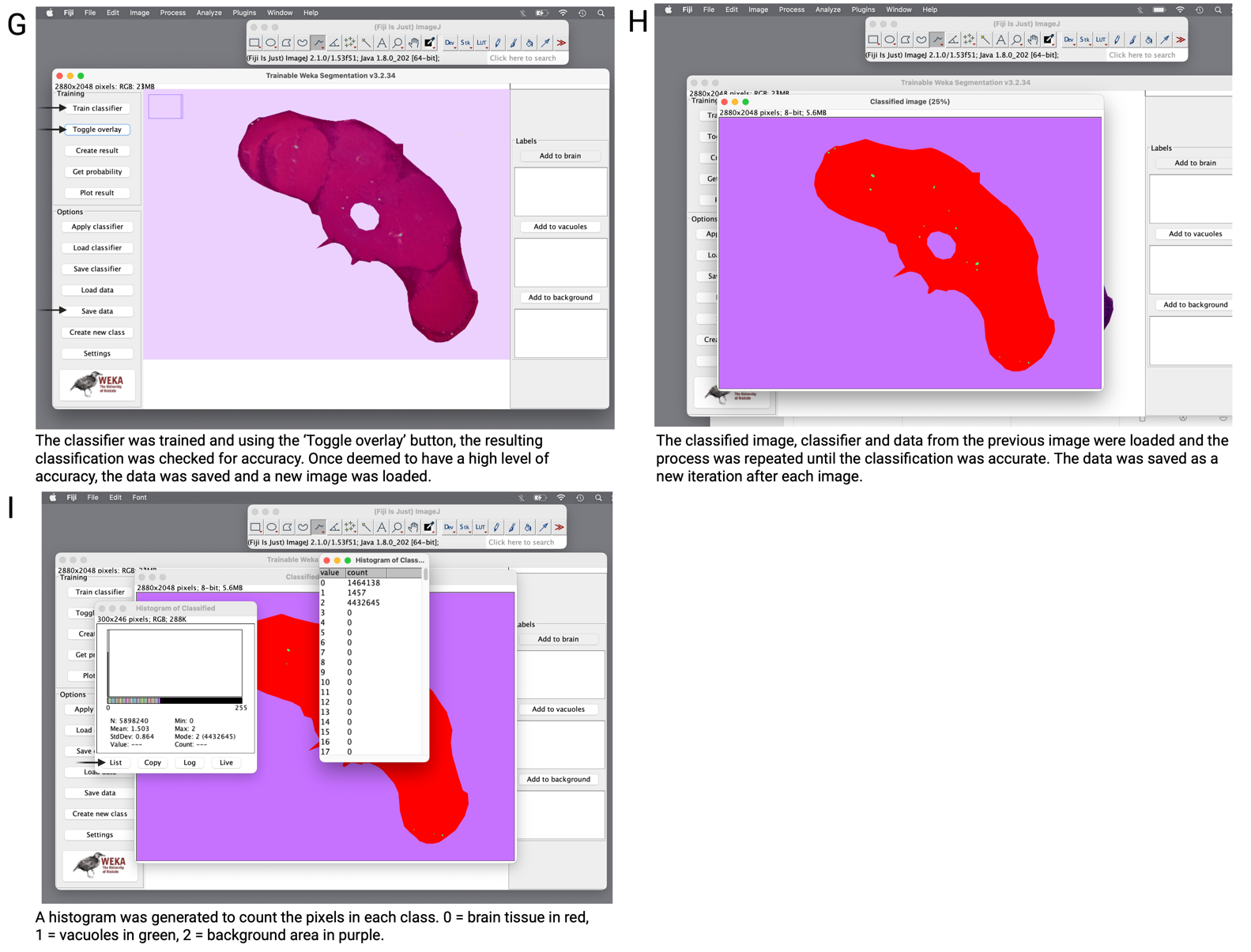
**

**Figure S1.** Using Fiji software, the Trainable Weka Segmentation (TWS) plugin (Arganda-Carreras et al 2017) can be trained to recognise and classify vacuoles and brain tissue, which can then be quantified by measuring the pixel counts in each class.

**
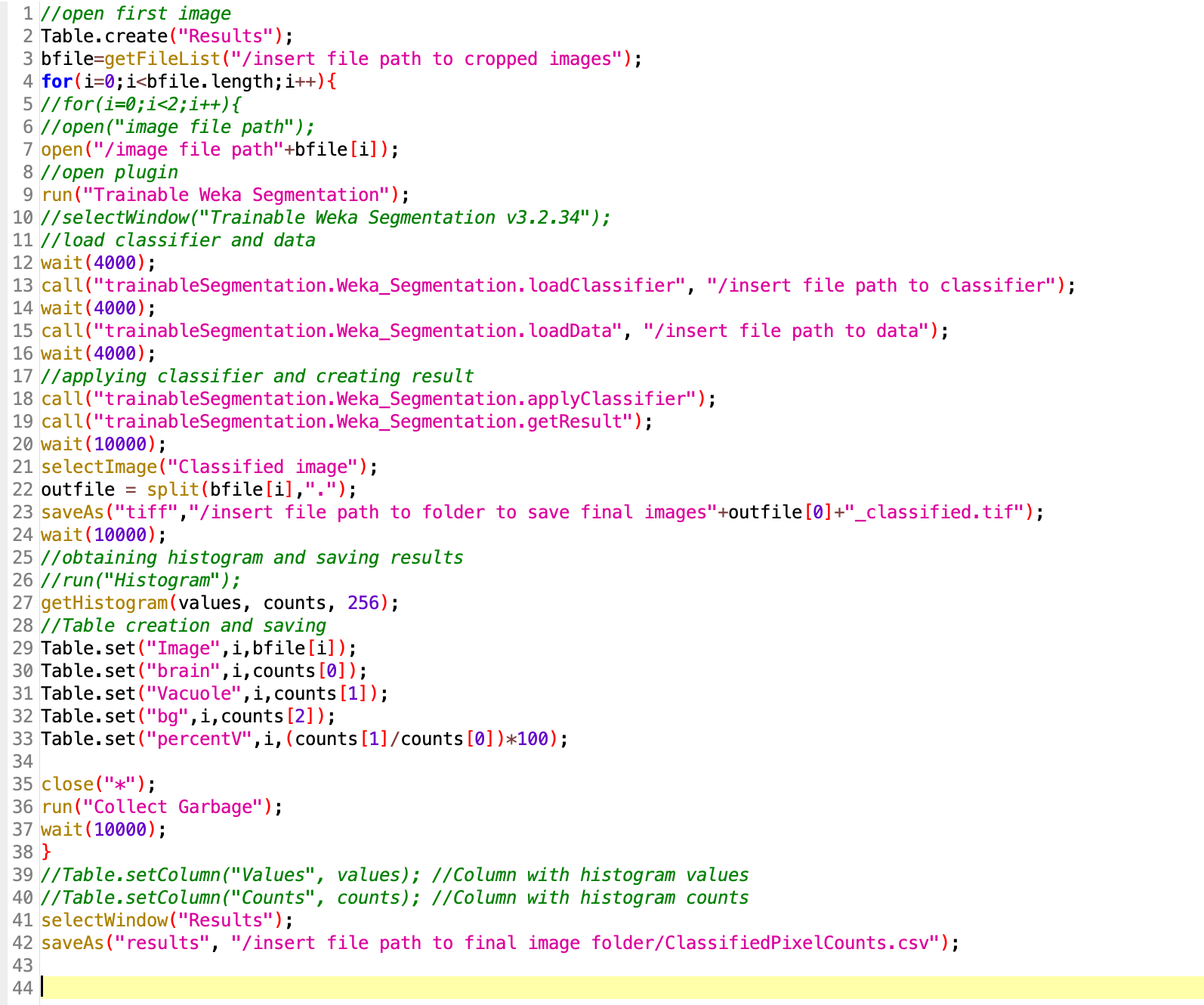
**

**Figure S2.** The VacQuant macro written in Fiji automates the classification and quantification of vacuoles in *Drosophila* brain tissue using the machine learning Trainable Weka Segmentation (TWS) plugin. The total area of vacuolation and brain tissue was calculated using pixel counts. The user may need to adjust the wait() values to suit their dataset.

#
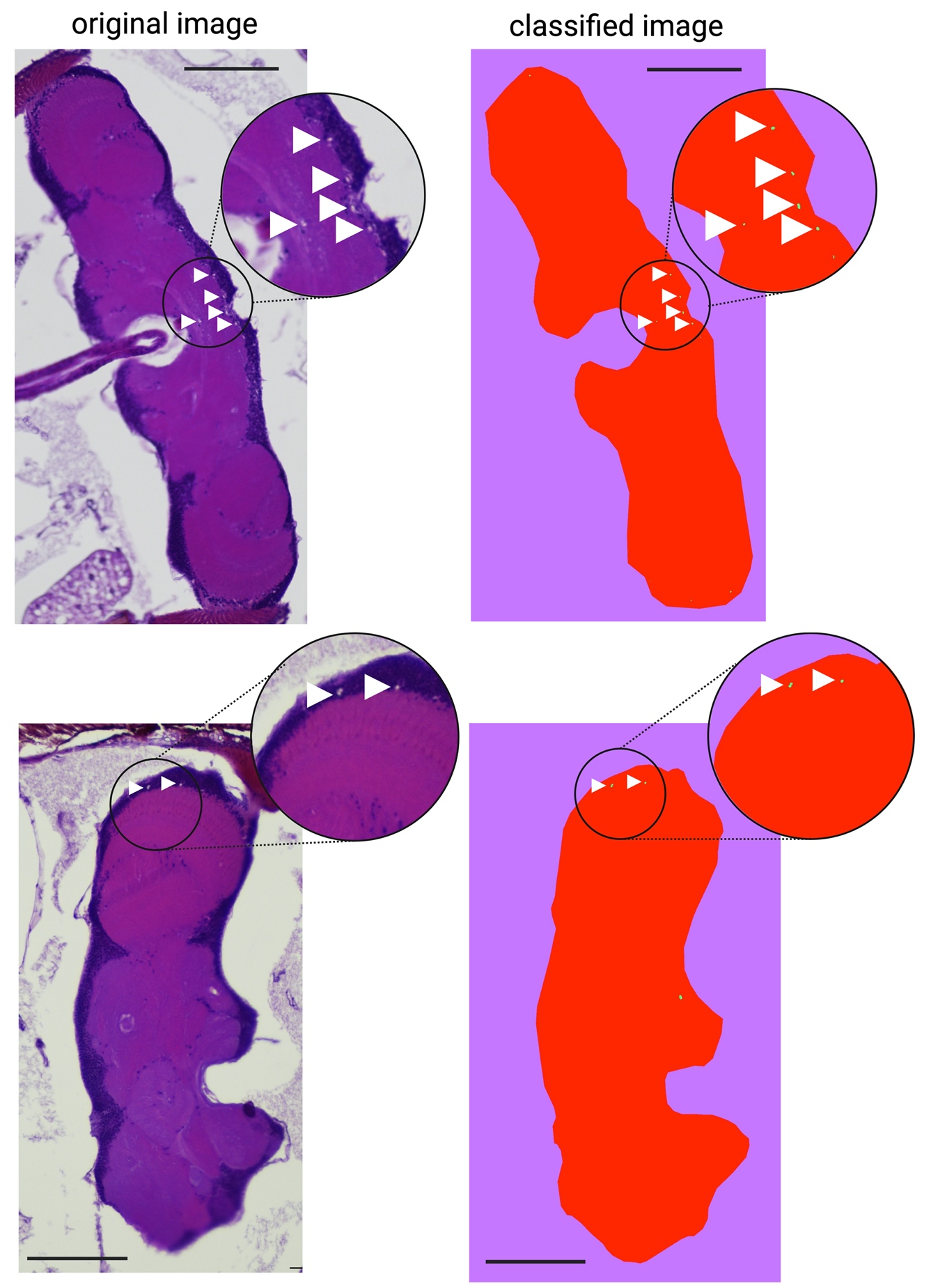
Figure S3. The Trainable Weka Segmentation (TWS) plugin can be trained to classify the pixels of an image of a *Drosophila* brain section stained with Haematoxylin and Eosin (H&E). The original images of individual brain sections from two control *Drosophila* aged 10 days post-eclosion are shown on the left. Following the protocol described in this paper, the TWS classifier was used to classify and quantify the pixels into brain tissue (red), vacuoles (green), and background (purple), and the resulting images are shown on the right. Vacuoles within enlarged area are indicated with white arrow head. Scale bar = 80 µm.

#
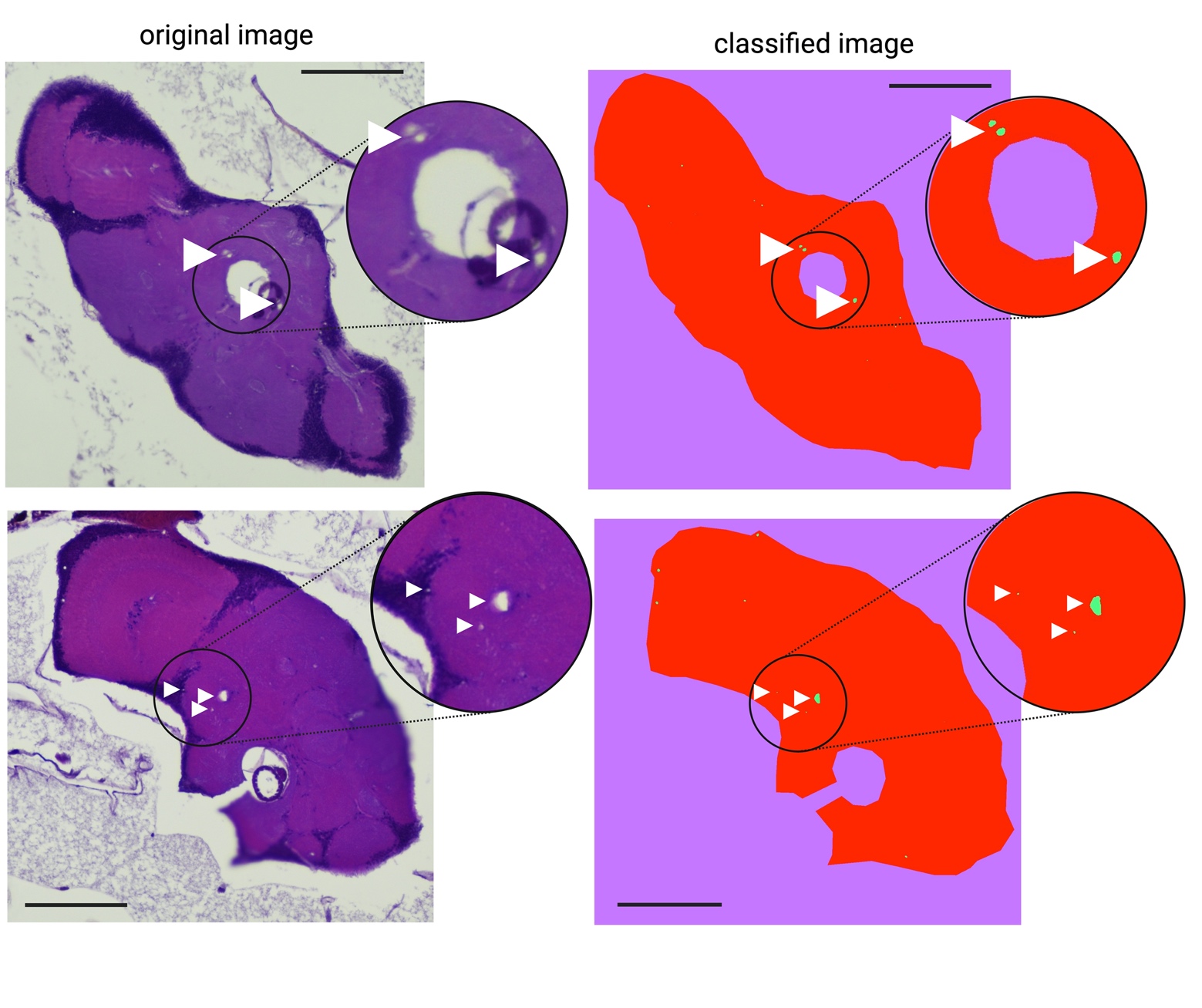
 Figure S4. The Trainable Weka Segmentation (TWS) plugin can be trained to classify the pixels of an image of a *Drosophila* brain section stained with Haematoxylin and Eosin (H&E). The original images of individual brain sections from two tau R406W expressing *Drosophila* aged 10 days post-eclosion are shown on the left. Following the protocol described in this paper, the TWS classifier was used to classify and quantify the pixels into brain tissue (red), vacuoles (green), and background (purple), and the resulting images are shown on the right. Vacuoles within enlarged area are indicated with white arrowhead. Scale bar = 80µm.

**
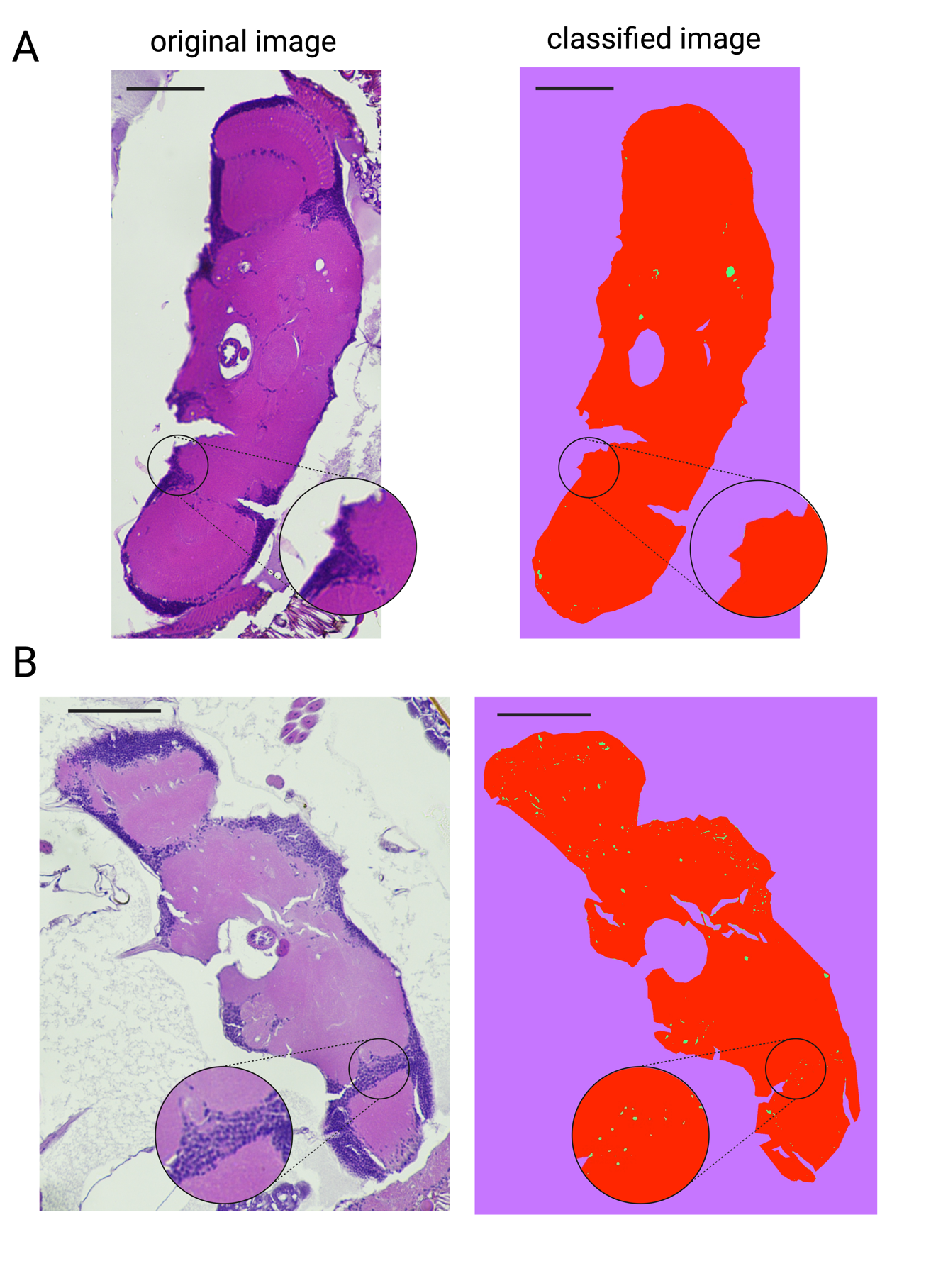
**

**Figure S5**. The original images of individual brain sections stained with Haematoxylin and Eosin (H&E) from two tau R406W expressing *Drosophila* aged 20 days post-eclosion are shown on the left. Following the protocol described in this paper, the TWS classifier was used to classify and quantify the pixels into brain tissue (red), vacuoles (green), and background (purple), and the resulting images are shown on the right. (A) An example of a double transgenic EGFP and tau R406W expressing fly brain section with large rips that have been successfully removed during the image cropping phase so that they do not affect the measurement of the total brain area of the tissue. (B) An example of a double transgenic Rab9 overexpressing and tau R406W expressing fly brain section with lighter staining. The enlarged areas clearly show that although the lighter stained section in B has successfully quantified a high number of vacuoles, when closely inspected there are areas of lighter staining that have been mistakenly detected as vacuoles. Meanwhile a similar area in brain section A without vacuoles shows darker staining and the TWS classifier has not detected vacuoles. Scale bar = 80µm.

# Troubleshooting Guide

- If the macro does not complete the current step before moving on to the next, add a ‘wait’ function of 4000 milliseconds – wait(4000). If it still moves on too quickly, increase the wait time.
- Make sure to turn off the sleep function on the computer. The macro will stop running if the computer goes to sleep.
- If the borders of the brain tissue and the background are incorrectly classified as vacuoles, crop closer to the brain tissue to remove the lighter stained edge. Alternatively, retrain the classifier, specifically selecting the border pixels and classifying them appropriately.
- Make sure a range of brain tissue areas are selected and classified during the training process to ensure the colour variations in the staining are accounted for and recognised.
- If a brain section spans two images, they can be combined into one image. Crop each image separately, making sure not to duplicate any brain regions, and then copy the brain region from the second image and paste it on to the first image. The classifier will then be able to calculate the full brain area and vacuolation as one quantification.

# References

Arganda-Carreras, I., Kaynig, V., Reuden, C., Eliceiri, K. W. Schindelin, J., Cardona, A., Seung, H. S. 2017. “Trainable Weka Segmentation: a machine learning tool for microscopy pixel classification.” Journal article. *Bioinformatics* 33(15):2424-2426. <https://doi.org/10.1093/bioinformatics/btx180>

Fischer, A. H., Jacobson, K. A., Rose, J., & Zeller, R. 2008. “Hematoxylin and eosin staining of tissue and cell sections”. *CSH protocols*, 1 (2008). <https://doi.org/10.1101/pdb.prot4986>.
